# Metal Oxide Nanoparticles: Evidence of Adverse Effects on the Male Reproductive System

**DOI:** 10.3390/ijms22158061

**Published:** 2021-07-28

**Authors:** Mariana Vassal, Sandra Rebelo, Maria de Lourdes Pereira

**Affiliations:** 1 Department of Biology, University of Aveiro, 3810-193 Aveiro, Portugal; marianavassal@ua.pt; 2 Neuroscience and Signalling Laboratory, Institute of Biomedicine (iBiMED), University of Aveiro, 3810-193 Aveiro, Portugal; 3 Department of Medical Sciences, University of Aveiro, 3810-193 Aveiro, Portugal; 4 CICECO-Aveiro Institute of Materials, University of Aveiro, 3810-193 Aveiro, Portugal

**Keywords:** metal-oxide nanoparticles, nanotoxicity, spermatogenesis, male infertility, reproductive system, oxidative stress, biomedicine

## Abstract

Metal oxide nanoparticles (MONPs) are inorganic materials that have become a valuable tool for many industrial sectors, especially in healthcare, due to their versatility, unique intrinsic properties, and relatively inexpensive production cost. As a consequence of their wide applications, human exposure to MONPs has increased dramatically. More recently, their use has become somehow controversial. On one hand, MONPs can interact with cellular macromolecules, which makes them useful platforms for diagnostic and therapeutic interventions. On the other hand, research suggests that these MONPs can cross the blood–testis barrier and accumulate in the testis. Although it has been demonstrated that some MONPs have protective effects on male germ cells, contradictory reports suggest that these nanoparticles compromise male fertility by interfering with spermatogenesis. In fact, in vitro and in vivo studies indicate that exposure to MONPs could induce the overproduction of reactive oxygen species, resulting in oxidative stress, which is the main suggested molecular mechanism that leads to germ cells’ toxicity. The latter results in subsequent damage to proteins, cell membranes, and DNA, which ultimately may lead to the impairment of the male reproductive system. The present manuscript overviews the therapeutic potential of MONPs and their biomedical applications, followed by a critical view of their potential risks in mammalian male fertility, as suggested by recent scientific literature.

## 1. Introduction

Nanotechnology is a field of science that studies the properties, design, manipulation, production, and applications of structures and devices at the nanoscale level (10^−9^ m). Objects on this scale, such as nanoparticles (NPs), have properties and functions that differ from those with a larger scale [1]. European and other International Committees have defined NPs, as particles of matter in which at least one of their phases has one dimension (length, width, or thickness) within the range of 1 to 100 nanometers (nm) [2,3].

Among the several types of NPs reported in the literature, metal oxide NPs (MONPs) stand out as the category of versatile materials. Being a type of metallic NPs that have controllable features and small size, making them able to easily cross cells and tissues within the body to reach a target location [4,5]. This makes MONPs a valuable tool for biomedical applications, such as anticancer, antidiabetic, antimicrobial purposes, as well as imaging applications, drug delivery, and even in reproductive medicine [6].

Most of these inorganic materials that make up MONPs are typically classified as biocompatible since their metallic precursors are already present in human tissues, whose vital role in body functions was reported [7,8]. Because they are essential to the body, they will be more readily accepted by the organism [6]. Manganese (Mn), molybdenum (Mo), magnesium (Mg), iron (Fe), cobalt (Co), chromium (Cr), copper (Cu), zinc (Zn), and selenium (Se) are among some of the elements considered essential for humans [6,7]. Some of these metals are closely related to male fertility. Zinc transduces a sign that induces sperm to become motile [9,10]. Selenium deficiency was previously associated with a decline in sperm motility [11]. Copper-dependent enzymes are present at all stages of spermatogenesis, as well as in somatic cells of the testis and epididymis [12]. However, in high concentrations, these physiologically compatible metals have toxic effects on mammalian cells and can even cause cell death [13]. Depending on how many metal ions are readily available, they can be beneficial or harmful, making their use a double-edged sword [6,12,14]. This may be part of the reason why there are so many controversial reports on the reproductive toxicity of MONPs [15]. In fact, it has been proven that MONPs can cross the blood–testis barrier (BTB), a structural and physiological compartment that protects spermatogenesis [16]. This raises concerns about male fertility, especially as spermatogenesis is a highly vulnerable process that is sensitive to exogenous materials, such as NPs [17,18]. Thus, addressing the effects of MONPs on the male reproductive system is crucial.

This review summarizes in vitro and in vivo studies that describe the potential reproductive toxicity of MONPs, to clarify the accurate effects of these NPs on the male reproductive system. Gaps in knowledge and ideas for future research are highlighted.

## 2. Classification of Nanoparticles and MONP Synthesis

**Figure 1:**
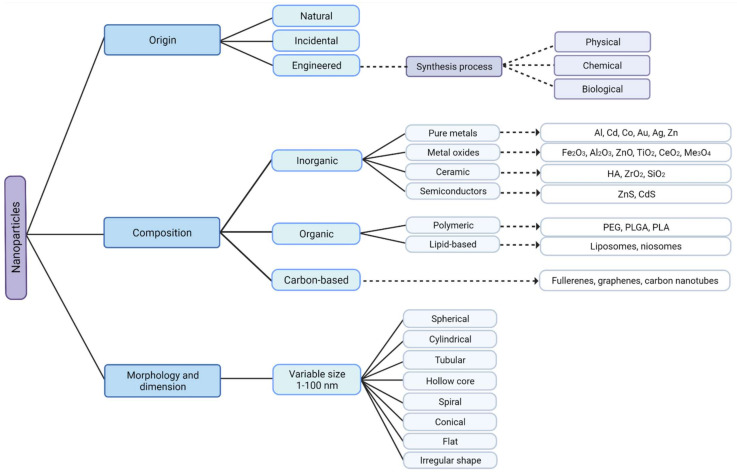
Classification of nanoparticles according to their origin, composition, morphology, and dimension with some examples. Metal oxide nanoparticles are engineered, inorganic nanoparticles, that can be synthesized by physical, chemical, or biological techniques, created with Biorender.com (accessed on 27 June 2021).

NPs are versatile nanosized structures and, therefore, can be classified according to their dimensions, morphology, materials properties, origin, and synthesis process (Figure 1) [19]. Regarding their classification, all NPs share some aspects: they are known to have reduced size, which is related to their high surface area to volume ratio, they have chemically alterable physical properties, easy surface functionalization, and they all have different physical properties in respect to the bulk material [5,20,21].

Based on morphology and dimensions, NPs are typically spherical, but they can have many other shapes, such as cylindrical, tubular, conical, hollow core, spiral, flat, or irregular in shape with variable size [22,23].

Nowadays, NPs can be produced incidentally because of human activities, as a by-product of industrial and domestic endeavors that result in the unintentional release of NPs into the environment. On the other hand, engineered NPs with new properties may be synthesized by rearranging atoms of an object. However, NPs are not entirely a product of modern technology. Some exist in the natural world and can be found everywhere on earth, that is, in the hydrosphere, atmosphere, lithosphere, and biosphere. Therefore, regarding their origin, NPs can be classified as incidental, synthetic/engineered, or natural [24]. This emphasizes the idea that nanotechnology has become even more pervasive, and that NPs are ubiquitous in the environment, becoming more deeply embedded in today’s life.

According to properties of their materials, engineered NPs can be classified as carbon-based if they are made completely of carbon (e.g., fullerenes, graphene, carbon nanotubes), metal-based if NPs are made purely from metal precursors (e.g., Al, Cd, Co, Au Ag, Zn), metal oxides based if they have been synthesized to modify the properties of their respective metal based NPs (e.g., Fe_2_O_3_, Al_2_O_3_, ZnO), ceramic NPs if they are nonmetallic solids (e.g., HA, ZrO_2_, SiO_2_) and semiconductor NPs if they have properties between metals and nonmetals (e.g., ZnS, CdS) [22]. Polymeric NPs (e.g., PEG, PLGA, PLA) and lipid-based NPs (e.g., liposomes, niosomes), unlike those just mentioned, are generally organic [3,19,25].

There is a broad variety of techniques that can be used to synthesize MONPs, each with its own advantages and disadvantages. Generally, they can be arranged into physical, chemical, and biological (green synthesis) methods [26]. Biologically synthesized NPs are preferred in biomedical applications since they are safer than those produced by traditional physicochemical approaches. This can be attributed to the fact that the metallic core of NPs is coated with non-toxic biomolecules, making them biocompatible [27]. Additionally, in this method, the use of dangerous substances, such as organic solvents and inorganic salts—which are commonly used in physical and chemical methods—is minimized [28].

However, unlike other methods, this green approach has the drawback of being unable to control the size, shape, and yield of NPs [29]. Essentially, no single technique is ideal in all aspects or for all applications. Therefore, the desired application should be considered to select the most appropriate method.

The exact physical and chemical properties of NPs depend on the different ways in which they are produced, namely the synthesis process, external factors (reaction temperature, concentration of reagents and type of capping agents used), and internal factors (morphology, size, concentration) during their production [30]. These parameters, in turn, will determine the interaction of NPs with biological systems [29].

## 3. Biomedical Applications of MONPs

MONPs are inorganic materials made to modify the properties of metallic elements. These have been subjected to intense biomedical research, mainly due to their unique intrinsic properties, such as good optical, electrical, catalytic, and magnetic behavior, chemical and mechanical stability, simple preparation process, easily engineered for the desired size, shape and porosity, and large surface area for reactions [4,5,31]. In addition, these materials can easily have their surface modified with several chemical functional groups, allowing their conjugation with antibodies, ligands and drugs of interest, which further enhances their potential in the biomedical field [5]. Although there is a wide spectrum of metals available, their use in the medical field is limited to those tolerated by the organism [32]. The fact that some metals exist in appreciable amounts in the body makes most MONPs biocompatible. For example, in the human body, iron (3–4 g) is mainly found associated with hemoglobin, making it the most abundant metal [33,34]. Followed by iron, zinc (~2 g) [35], and copper (~0.1 g) [36] are the second and third most common metals in the human body, and they are essential constituents of several enzymes. Unlike the previous metals, manganese is present in very small amounts in the body (~12 mg). However, it is one of the most important nutrients for human health as it assists in the development of connective tissue, bones, blood-clotting factors, and sex hormones [33].

The use of MONPs to treat cancer, diabetes, and even to eradicate infectious diseases has been extensively studied, which proves the effort that has been made to create a symbiosis between nanoscience and medical science [31,37].

**Figure 2:**
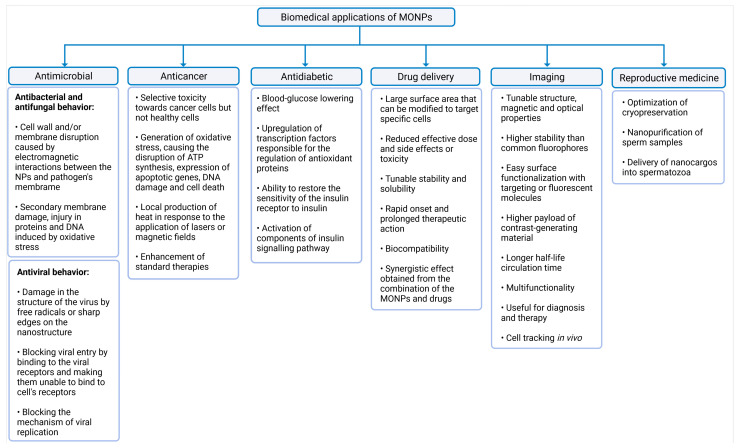
Summary of the biomedical applications of MONPs. The latter were divided into six categories, namely antimicrobial activity, anticancer activity, antidiabetic activity, drug delivery, imaging, and reproductive medicine, created with Biorender.com (accessed on 27 June 2021).

The common biomedical applications of MONPs and their main mechanisms of action are summarized in Figure 2.

### 3.1. Antimicrobial, Anticancer, and Antidiabetic Activity

Although in excessive doses many metals are toxic to all cell types, in lower concentrations, MONPs may be able to selectively target bacteria, since their metal transport system and metalloproteins are different from those existing in mammalian eukaryotic cells [38,39]. To exert this microbial function, MONPs need to be in contact with microbial cells. This interaction increases microbes’ membrane permeability, and allows the entry of NPs into the cytoplasm [38,40], where NPs induce damage to cellular macromolecules (Figure 2) [41]. This antimicrobial activity is enhanced for higher concentrations and smaller MONPs sizes [42,43], since smaller sizes allow a closer contact between NPs and the microbial membrane [4].

A wide range of MONPs seem to have antimicrobial abilities, including titanium dioxide (TiO_2_) [44], magnesium oxide (MgO) [45], zinc oxide (ZnO) [46], copper oxide (CuO) [47], iron oxide (Fe_3_O_4_) [48], cerium oxide (CeO) [49], and silver oxide (Ag_2_O) [50]. The molecular mechanisms of the antifungal activity of MONPs have been less studied because most studies have focused on antibacterial activity. Nonetheless, recent research suggests that these MONPs have similar mechanisms for bacteria and fungi [51].

Besides presenting antibacterial and antifungal activities, some MONPs also exert antiviral properties (Figure 2). MONPs can adhere to the virus envelope, causing its destruction [52], or they can block their mechanism of viral replication [53] or viral entry into a cell [54]. Metal oxides, such as TiO_2_ [52] and Cu_2_O [55], have already been shown to be effective antiviral agents against influenza A virus subtype H3N2 and Hepatitis C, respectively. These findings open a new perspective to prevent and treat viral diseases using MONPs.

MONPs can also selectively target cancer cells [56] and exert their anticancer activity mainly through the generation of oxidative stress [57]. This property can be further enhanced with the application of external stimuli such as magnetic fields or lasers, which induce the local production of heat in tumor sites [58]. Additionally, these NPs can also be used as enhancers of standard therapies, acting as co-adjuvants to improve the effect of radiation on radiotherapy, or to facilitate the action of conventional anticancer drugs, reducing the required dose and side effects of such drugs [59]. Therefore, different strategies take advantage of MONPs in the treatment of cancer: alone, conjugated with biological molecules, ligands, and anticancer drugs, or in combination with other conventional therapies to potentiate their therapeutic efficacy [60].

In addition, other MONPs such as MgO, MnO [61], CeO_2_ [62], ZnO [63,64], and Fe_2_O_3_ [65] have been explored as possible antidiabetic agents, since recent studies have shown promising results. Essentially, the antioxidant ability of MONPs contributes to a decrease in oxidative stress, which is the main cause of β-cell damage [66]. However, concentration determines whether NPs elicit oxidative stress or increase the cell antioxidant capacity. Generally, small doses seem to be related to the antidiabetic potential [14,65].

### 3.2. Drug Delivery Platforms and Imaging

Medical imaging is essential for medical diagnosis. MONPs have been used as nanoparticle-based contrast agents in multiple modern imaging modalities that allow the visualization of abnormalities, such as tumor lesions or other regions of interest [67]. Of all the plethora of available NPs, metal oxides have advantages in imaging applications due to their diverse size- and shape-dependent optoelectronic properties [27,68] and high stability, which are not achievable with traditional lipid or polymer-based nanoparticles [69]. In addition, compared to molecular probes, MONPs are virtually inert, which means that they hardly interact with other cellular molecules and, therefore, their optical properties remain unaffected [70]. Their surface can also be easily functionalized with drugs, targeting or fluorescent molecules, or other components [71,72]. Therefore, these contrast agents can deliver therapeutic agents simultaneously, allowing for a dual diagnostic and therapeutic effect [73].

Considering all this, MONPs are attractive imaging agents. As a result, they have been exploited for different imaging modalities, such as magnetic resonance imaging (MRI) [74], photoacoustic imaging (PA) [75], positron emission tomography (PET) [76], computed tomography (CT) [77], fluorescent imaging [78], among many others. In addition, NPs can be multifunctional and, therefore, can provide contrast for more than one imaging modality [23].

### 3.3. An Asset for Reproductive Medicine

Although the detrimental effects of NPs on male fertility and sperm cell function have been suggested [16], some research teams have been exploring the properties of these materials to improve assisted reproductive techniques. Falchi et al. reported that the incubation of ram semen with CeO_2_ NPs during cryopreservation improved sperm quantity and quality [79]. This study suggests that CeO_2_ NPs can have beneficial effects on sperm preservation. Other research teams have functionalized Fe_2_O_3_ NPs with lectins and antibodies, to selectively bind to glycans expressed in acrosome reaction, or to ubiquitin, which is present on the surface of defective spermatozoa [79,80]. Then, aberrant spermatozoa can be removed from a sample using a magnetic force. This method of sperm purification may be used to increase conception rates following artificial insemination [80]. Nanoplatforms for the delivery of biological compounds to spermatozoa are another nanotechnology that has been investigated in the field of reproductive medicine [15].

Makhluf et al. described the spontaneous penetration of polyvinyl alcohol (PVA)-Fe_3_O_4_ NPs in bovine sperm, without affecting their motility and ability to undergo the acrosome reaction [81]. These interesting results suggest that, in the future, NPs may be conjugated with target nutrients or treatments for direct nutrient supplementation to sperm.

These and other research teams have presented interesting results that highlight the usefulness of MONPs. However, despite these promising results, uncertainty remains about the safety of MONPs. Therefore, it is crucial to investigate in more detail how MONPs interact with the male reproductive system and the consequences of this exposure.

## 4. The Impact of MONPs on Male Fertility

MONPs have received a lot of attention, especially in the biomedical field, due to their biological usefulness, as discussed in previous sections. In addition, due to their unique properties and versatility, the application of NPs extends to many other fields, making them ubiquitous in the environment. Consequently, human exposure to nanomaterials has increased dramatically. However, in recent years, the use of NPs of any material has become controversial [82]. On one hand, MONPs can interact with cellular macromolecules, leading to therapeutic effects [83]. On the other hand, cytotoxic effects were found in some tissues, presenting a health hazard [84].

Many studies suggest that human male infertility has increased significantly over the past few decades [85,86,87]. Due to this alarming trend, it has been hypothesized that environmental, dietary, and/or lifestyle changes are interfering with men’s ability to produce spermatozoa with a consequent impact on male fertility [88,89]. In addition, the male reproductive system is known to be susceptible to environmental stress, as toxicants, vehicular pollutants, and even NPs [90]. As a result, the impact of MONPs on male reproductive health has become an important subject of study. While several reports suggest that some NPs might have protective effects on sperm cells [91], other reports suggest that they compromise male fertility by interfering with spermatogenesis [92]. In fact, spermatogenesis is prone to errors. Defects in any of its steps can result in the failure of the entire process and, in some cases, can lead to testicular diseases or male infertility [93,94].

**Figure 3:**
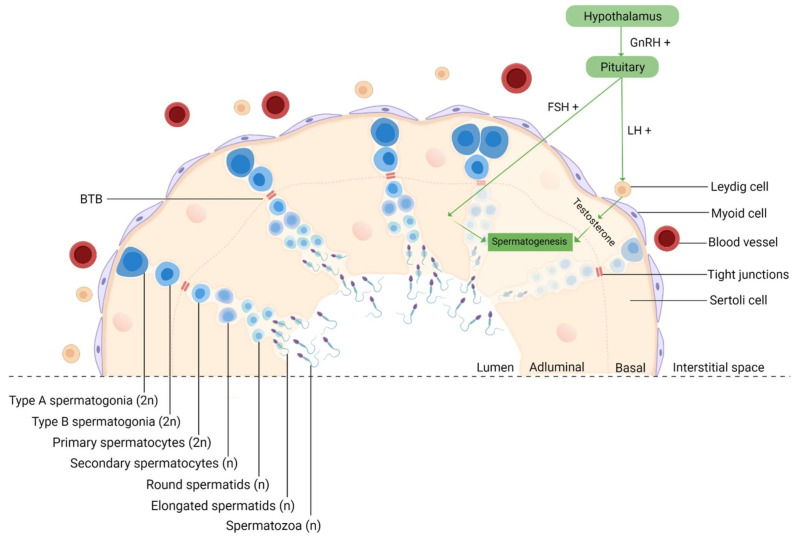
Schematic representation of spermatogenesis in the cross-section of a seminiferous tubule. Spermatogenesis is initiated at puberty by the hypothalamus, which produces GnRH, which, in turn, stimulates the release of FSH and LH at the reproductive tract. LH stimulates Leydig cells to produce testosterone and FSH stimulates Sertoli cells that provide support and nutrition for sperm survival, proliferation, and differentiation [102]. Sertoli cells then initiate the functional responses required for spermatogenesis. Spermatogenesis starts when type A spermatogonia (2n) commit to differentiating into type B spermatogonia. Then, through mitosis, B-spermatogonia (2n) give rise to primary spermatocytes (2n). The latter undergo a long meiotic phase that originates the secondary spermatocytes (n), which ends with spermatids (n) generation [103]. The round spermatids then go through substantial morphological changes during spermiogenesis originating highly specialized spermatozoa through the reorganization of the entire cell, where the nuclear envelope seems to be crucially involved [104,105]. The next event is spermiation, in which mature spermatids are released from the supporting Sertoli cells into the lumen of the seminiferous tubule, and the remainder of the spermatid cytoplasm, known as the residual body, is phagocytosed by the Sertoli cells [106]. However, at this stage, spermatozoa still lack motility. Immotile spermatozoa are then transported into the epididymis where the final steps of maturation occur [107]. GnRH, gonadotropin-releasing hormone; LH, luteinizing hormone; FSH, follicle-stimulating hormone; BTB, blood–testis-barrier; 2n, diploid cell; n, haploid cell, created with Biorender.com (accessed on 2 July 2021).

Since spermatogenesis is a highly vulnerable process, it occurs in a protected environment, controlled by the BTB, whose purpose is to protect the developing germ cells from external insults [17]. It is formed by tight junctions between Sertoli cells that divide the epithelium of the seminiferous tubules (ST) into two different compartments: basal and adluminal (Figure 3). Although it is one of the tightest blood–tissue barriers in the mammalian body [95], it was previously reported that NPs could cross this biological barrier due to their ultra-small size [16]. In fact, in mice treated with TiO_2_ [96] and Fe_2_O_3_ [97], both NPs were able to penetrate the testis, despite the BTB. Takeda et al. even reported that TiO_2_ NPs accumulated in the testis of male offspring from pregnant mice who were treated with these NPs [98]. Other animal studies have also demonstrated that NPs can move from the initial absorption site, for example, the lungs and skin, to secondary organs, such as the testis [99]. The integrity of BTB is a concern since NPs can easily permeate cells and their nuclei. This creates favorable circumstances for mutations appearance, which in germ cells may interfere with fertilization, embryogenesis [100], or even generate congenital defects in the offspring [101].

Therefore, a clear understanding of the impact of MONPs on reproductive health is fundamental. Table 1 and Table 2 summarize the adverse effects of different MONPs on the male reproductive system, both in vitro and in vivo. However, it is important to keep in mind that these effects depend on several factors, such as dosage, duration of exposure, administration route, chemical nature of the compound (e.g., method of synthesis, size, shape, surface charge), as well as the biological system involved (e.g., strain and age of animal/cell, cell variability) [15].

### 4.1. In Vitro Studies

Few studies have focused on the adverse effects of NPs on male germ cells in vitro (Table 1).

The summary studies provide valuable information on the outcome of the interaction between MONPs and germ cells, which is useful for establishing the mechanisms of MONP toxicity. Parameters such as cell viability, oxidative stress, DNA damage, nanoparticle internalization, and mechanisms of cell death were assessed.

The in vitro studies reported in Table 1 were carried out with NPs made from Cerium (Ce), Iron (Fe), Manganese (Mn), Titanium (Ti), and Zinc (Zn) oxides. However, TiO_2_ and ZnO NPs are, by far, the most explored NPs.

The studies listed were conducted in different reproductive cells at three stages of maturation: spermatogonia, spermatocyte, and spermatozoa. Additionally, the cells responsible for testicular architecture and function, namely Sertoli and Leydig cells, were also used in the listed studies. In addition, most studies have carried out the extensive chemical and physical characterization of NPs, which is crucial for a better understanding of the toxicity mechanisms of NPs on reproductive cells.

A wide range of concentrations of MONPs has been studied, from very low (0.04 µg/mL) to high concentrations (1000 µg/mL). It is crucial to evaluate different concentrations of MONPs to establish their cytotoxic effect. However, the results still were conflicting. Préaubert et al. reported that the lowest concentrations of CeO_2_ NPs (0.01 µg/mL) were associated with higher levels of DNA damage in human spermatozoa [108]. ZnO NPs were also highly cytotoxic to mouse Leydig cells, even at low concentrations and incubation times [117]. Those are the exceptions since most studies indicate that MONP cytotoxicity is dose and time-dependent. Other authors even reported that lower MONPs concentrations were inefficient to cause genotoxicity [92,111].

The periods of incubation were also variable, ranging from 15 min to 24 h. From the results summarized in Table 1, it can be deduced that the reproductive toxicity of MONPs depends mainly on the concentration used and on the time of incubation.

The size of the NPs used ranges from ultrafine particles (7 nm) to much larger NPs (177 nm). Previous studies reported that even a small difference in size can make particles up to six times more harmful [119]. Gromadzka-ostrowska et al. also found that the toxicity of NPs is not only dependent on dose and time, but also depends on size, which seems to be inversely proportional to the cytotoxicity of NPs [120]. However, none of the studies reported in Table 1 evaluated the effect of the size of NPs on male germ cells.

The most studied parameters were oxidative stress indexes, cell viability, apoptosis, and genotoxicity. The principal suggested mechanism by which MONPs may exert that their toxic and genotoxic effect is oxidative stress [113,117]. In fact, increased oxidative stress was observed in almost all studies where this parameter was tested, except one [117]. Bara and Kaul reported an increase in the levels of antioxidant enzymes SOD and CAT in Leydig cells after exposure to ZnO NPs [117]. However, it has also been reported by other studies that NPs initially induce antioxidant enzyme activities in response to stress, as a defense mechanism, but, eventually, ROS production overcomes the capacity of the antioxidant response mechanisms [121].

**Figure 4:**
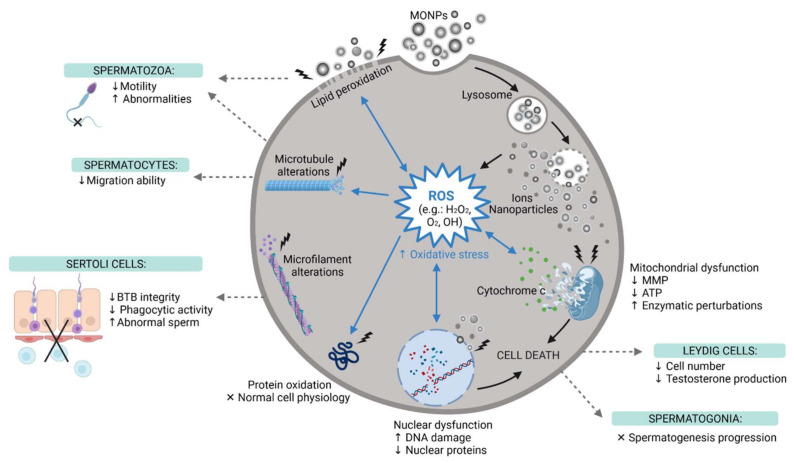
The main reproductive toxic events induced by MONPs at the cellular level. MONPs, Metal Oxide Nanoparticles; ROS, Reactive Oxygen Species; MMP, Mitochondria Membrane Potential; ATP, Adenosine Triphosphate; BTB, Blood-Testis-Barrier; ↑, increase; ↓, decrease; x, impaired, created with Biorender.com (accessed on 30 May 2021).

Both exogenous stimuli and endogenous physiological stress can induce ROS production [117]. Oxidative stress is known to induce DNA damage through the oxidation of DNA bases [108] (Figure 4). However, it can also induce injury to biomolecules and organelles in other cells, mainly mitochondria [117]. In addition, under stress conditions, cells activate different cellular processes important for cell adaption to adverse conditions or to activate cell mechanisms of cell death, such as apoptosis or necrosis [117]. Pinho et al. reported an increase in the number of spermatogonia in necrosis (but not apoptosis) after ZnO NP exposure [92], while other studies have reported apoptosis as the preferred mechanism of cell death [110,117,118]. Autophagy is an example of an adaptive mechanism under stress conditions, and it was reported in Leydig cells after ZnO NPs exposure [118].

The mechanism of MONPs internalization by cells was explored in some studies. Pawar and Kaul, using Scanning Electron Microscopy (SEM) and Transmission Electron Microscopy (TEM) images, reported that TiO_2_ in both agglomerated and single forms can remain attached to the spermatozoon surface (head and tail) after the addition of NPs to the sperm suspension, even after washing [111]. This indicates that NPs can attach and remain intact on the cell membrane immediately after mixing the NPs with the cell suspension. When in direct contact with cells, NPs cause mechanical damage to the membrane and destabilization of the plasma membrane, allowing NP entrance. The latter will exert pro-oxidant effects. In fact, Mao et al. monitored the internalization of TiO_2_ NPs by spermatocytes and Sertoli cells, both by flow cytometry and by TEM [112]. Bara and Kaul TEM results also revealed that ZnO NPs can enter Leydig cells and cross their nuclear membranes [117]. Moreover, Préaubert et al. also found an accumulation of CeO_2_ NPs at the spermatozoon plasma membrane [108]. However, in this case, the NPs were not internalized by the cells, but genotoxicity was still present. These authors proposed that MONPs do not need to be internalized to induce cell damage. To date, the exact mechanism by which the NPs induce cell damage is far to be elucidated, and, therefore, more comprehensive studies are needed.

Changes in the cytoskeleton were assessed only by Mao et al. and Pinho et al., using TiO_2_ NPs and ZnO NPs, respectively [92,112]. The latter reported disturbances in both microtubules and microfilament networks in spermatogonia cells [92]. Mao et al. also studied the effect of TiO_2_ NPs on the cytoskeleton of two different germ cells, spermatocytes, and Sertoli cells. TiO_2_ NPs interfered with microtubules of spermatocytes, but Sertoli cells only had their microfilaments altered [112]. These studies indicate that different germ cells respond differently to MONP insults. Additional studies should investigate alterations in the cytoskeleton since changes in the microtubule dynamics affect the formation of sperm flagella and migration abilities, and changes in the microfilament dynamics can affect the formation of tight junctions of Sertoli cells, which altogether interfere with spermatogenesis [112]. Although Liu et al. did not study cytoskeleton dynamics, their results indicate downregulation of tight junction proteins in Sertoli cells, leading to BTB impairment [116]. In addition, the disturbed microfilament arrangement interferes with the phagocytic capacity of Sertoli cells, which makes cells unable to properly phagocytose abnormal sperm cells [112].

Besides studying the cytoskeleton, Pinho et al. also reported, for the first time, the impact of ZnO NP exposure in the nucleoskeleton [92]. These authors reported several nuclear alterations in spermatogonia that may affect the progression of spermatogenesis.

Bara and Kaul was the only in vitro study to investigate the effect of NPs on steroidogenesis and testosterone biosynthesis in male reproductive cells. Interestingly, they found that a low concentration treatment with ZnO NPs for short incubation periods enhanced the steroidogenic ability of Leydig cells [117]. However, the exact mechanism is still unclear and therefore should be explored in future studies.

Overall, the interesting data collected indicate that the reproductive toxicity of NPs is not simply a matter of the NP material type, size, concentration, and exposure time, but also the result of intricate interactions at the nano-bio interface, which is influenced by many factors [13].

Since in vitro studies cannot consider tissue distribution, organs accumulation, clearance, or diffusion across biological barriers, such as the BTB, in vivo studies must be considered [122] and are of paramount importance to understand NP cytotoxicity.

### 4.2. In Vivo Studies

Table 2 lists the biochemical, molecular, and histopathological evidence of reproductive toxicity of MONPs. All MONPs that have been used in previous in vitro studies were also applied in in vivo studies. Considering the aluminum oxide (Al_2_O_3_) NPs, they have not been evaluated under cell culture conditions, only in vivo.

Table 2 clearly shows that animal models used for the in vivo experiments were mice and rats of different strains. Most of the studies listed have addressed the toxicity of MONPs at concentrations that are far from real-life conditions, even though there is no information available on the concentration of MONPs to which humans are exposed. Lauvås et al. used a lower and more realistic intratracheal dose of TiO_2_ NPs (63 µg/week for seven weeks), based on the estimated lung deposition of titanium at the Danish occupational exposure limit [137].

The exposure times used for the studies were highly variable. In some studies, male mammals received MONPs for very short periods, like 4 days [126], and, in other studies, the MONPs were used for much longer periods, namely six months [131]. These studies with these differences in exposure times are crucial since they help to create a better understanding of the acute and long-term effects of MONP administration. Additionally, many experiments have established the duration of treatment at around four weeks, to accomplish the duration of complete spermatogenesis in mice and rats [149].

Different routes of MONPS administration were used in animal experiments, namely, oral, intragastric, intratracheal, intraperitoneal, intravenous, and subcutaneous administration. It has been previously reported that there is very low absorption of MONPs through inhalation or oral administration in animals [62]. This aspect was confirmed by Lauvås et al., which was the only study included in Table 2, which administered MONPs intratracheally, and found that sperm cells are not susceptible to MONP exposure via the airways, at low doses [137]. On the other hand, in oral exposure, MONPs release more ions in the stomach due to the acidic environment. Therefore, this dissolution may be a reason for the cytotoxicity reported in the studies that used this administration approach, although fewer amounts of NPs are absorbed [145]. In contrast, intraperitoneal injections ensure proper absorption of the tested MONPs due to the highly vascularized peritoneal cavity [140]. The intravenous administration of nanomaterials ensures a much higher direct testicular exposure since NPs are administered directly into the bloodstream.

Regarding the parameters observed, most studies measured the weight of the male reproductive organs. Only Tang et al., Yousef et al. and Radhi et al. reported its increase after the oral administration of NPs, which may be attributed to the inflammation and hypertrophy or even accumulation of NPs in those tissues [90,123,147]. In fact, all studies that evaluated the content of MONPs in the testis and epididymis confirmed their presence in these organs. This was the case for cerium [124], iron [97], manganese [110], titanium [131,134,138], and zinc [90] NPs. The only exception was reported by Miura et al. studies, in which TiO_2_ NPs administered intravenously were found in the testis, but not in significant amounts [134,138]. This deposition of NPs in the reproductive tissues triggers the harmful events that will be described throughout this section. In fact, the damage has been reported in the testis and epididymis. Al_2_O_3_ [123], F_2_O_3_ [125], Fe_3_O_4_ [126], Mn_3_O_4_ [110,128], MnO_2_ [129], TiO_2_ [131,132,135,136,139,140], and ZnO [123,140,141,143,144,146] NPs all caused histopathological changes in the testis, mainly due to degeneration of the seminiferous tubules. Furthermore, Morgan et al. studied the histopathological changes induced by TiO_2_ NPs in the prostate and seminal vesicle, and reported that these reproductive organs were also affected by NPs, since they caused congestion, hyperplasia, and desquamation of the prostate’s epithelial, lining, and congestion in the seminal vesicle [133]. Salman also reported that ZnO NPs caused mild damage in seminal vesicles but severe damage to the prostate [148]. The reduction in the testis cell population has also been commonly reported, which is an indicator of a lack of active spermatogenesis in the testis [150].

The translocation of MONPs from their site of administration to the testicular tissue confirms that these NPs can cross and enter the BTB, where they interfere with normal physiological processes. Then, when in contact with reproductive tissues, these NPs can permeate cell membranes, inducing the overproduction of ROS, which leads to oxidative stress (Figure 4). This interferes with the balance between the oxidant and antioxidant systems, which causes oxidative damage in biomolecules, such as lipids, proteins, and nucleic acids [97]. To confirm the oxidative damage caused by MONPs, different studies evaluated ROS production and the levels of other oxidant markers, such as Malondialdehyde (MDA), Nitric Oxide (NO), Protein Carbonyl Content (PC), Lipid Peroxidation (LPO), and Total Oxidant Status (TOS). Antioxidant parameters such as Superoxide Dismutase (SOD), Glutathione Peroxidase (GPx), Reduced Glutathione (GSH), Catalase (CAT), and Total Antioxidant Capacity (TAC), were also evaluated. These parameters of oxidative stress were assessed on all types of MONPs, except CeO_2_ NPs [124]. The results reported an increase in oxidant markers and a decrease in intracellular antioxidant defenses and TAC. This confirms that MONPs suppress the antioxidant machinery and induce oxidative stress, which can lead to various cellular damages and, consequently, interfere with male fertility. In fact, according to previous studies, 30–80% of male infertility cases can be attributed to oxidative stress-mediated injury to the male reproductive system [110,151,152]. Persistent oxidative stress leads to the downregulation of Bcl-2 and upregulation of Bax, which results in the leakage of cytochrome c from dysfunctional mitochondria, ultimately resulting in apoptosis (Figure 4), through the activation of caspase molecules, as confirmed by Sundarraj et al. Meena et al., Shen et al. and Morgan et al. [97,118,132,135]. MONPs not only induce apoptosis, but some have also proven to be autophagy activators and inducers of autophagic cell death [118].

The levels of endocrine and reproductive hormones were also evaluated, and the results also suggest an imbalance in reproductive hormones (Testosterone, FSH, LH, GnRH, E2) and thyroid hormones (TSH, T3, T4) that can be attributed to the increase of ROS and the concomitant reduction of antioxidant enzymes. The exceptions were Lauvås et al. and Ogunsuyi et al., who reported that TiO_2_ NPs did not trigger alterations in testosterone levels [137,140]. Contrarily, Miura et al. reported that TiO_2_ NPs affected testosterone levels, but not FSH, LH, and GnRH [134].

Additionally, some authors explored the influence of MONPs on the expression of genes related to steroidogenesis. Testosterone is produced mainly in Leydig cells by a series of enzymatic reactions. First, the StAR protein transfers cholesterol to mitochondria. Then, the mitochondrial cytochrome P450scc transforms cholesterol into pregnenolone. Subsequently, other enzymes (3β-HSD, P450c17, 17β-HSD) convert the pregnenolone into testosterone [124]. Interestingly, Nr5A1, a transcription factor that regulates the expression of steroidogenic genes in Leydig cells (such as 3β-HSD), was downregulated after exposure to ZnO NP [144]. The StAR protein was also downregulated by CeO_2_ [124] and ZnO NPs [90], which can manifest in their inability to transfer cholesterol to the inner mitochondrial membrane, which stops steroidogenesis and justifies the decline in testosterone levels in most of the results listed. However, Bara and Kaul reported the conflicting results of increased testosterone production and StAR upregulation, but this was only related to small concentrations of ZnO NPs [117]. Ogunsuyi et al. did not report alterations in testosterone levels after intraperitoneal administration of TiO_2_ NPs; however, these levels were increased in the same study, under the same conditions, by ZnO NPs [140]. Likewise, Lauvås et al. found no significant alterations in testosterone levels after intratracheal administration of TiO_2_ NPs [137].

Sperm parameters, such as sperm number, viability, abnormalities, and motility, have been extensively studied. All studies that analyzed sperm count observed its decline with increasing concentrations of NPs, except for Varzeghani et al., Lauvås et al. and Song et al., who did not report significant alterations [126,136,137]. The results listed in Table 2 also indicate a reduction in motile spermatozoa, which affects their fertilizing potential. This decrease in sperm motility may have been a result of lipid peroxidation [140] (Figure 4). In addition, Morgan et al., Hussein et al., Srivastav et al. and Abbasalipourkabir et al. were the only research teams that evaluated sperm viability, having reported its decline [133,135,142,144,145]. An increase in sperm abnormalities, such as small head, double head, formless head, and double tails, has also been reported, which may be the result of oxidative damage [140] (Figure 4). These results are in agreement with those reported under in vitro conditions (Table 1).

Hong et al. evaluated the activity of metabolism-related enzymes—LDH, SDH, and SODH—that play key roles in the growth and development of testicular cells [130]. The results suggest that there was a decline in their activity, which may be associated with the disturbance of energy metabolism in germ cells. It was also the only study to evaluate the testicular activity of G-6PD, testis-marker enzymes ACP and AKP, and the activity of Ca^2+^-ATPase, Ca^2+^/Mg^2+^-ATPase and Na^+^/K^+^-ATPase. G-6PD is associated with androgen biogenesis, and its reduction implies that TiO_2_ NPs interfered with androgen secretion. In this study, ACP and AKP were used as markers of impaired spermatogenesis. Since ACP is related to the degeneration of the seminiferous epithelium and AKP is related to the activity of division of germ cells, their increase suggests testicular degeneration. Reductions in ATPases suggest an imbalance in the concentrations of intracellular ions, which could promote spermatogenesis dysfunctions [130].

Due to their small size, MONPs can reach the nucleus and interact directly with DNA, which causes the generation of ROS that further damages DNA (Figure 4) [146]. Not all studies tested the genotoxicity of NPs, but all studies that evaluated DNA damage later confirmed it. Mesallam et al. detected DNA fragmentation in the testis and prostate of rats treated with 422 mg/kg ZnO NPs daily for four weeks [146]. Meena et al. also found DNA strand breaks in spermatozoa of rats treated with 25 and 50 mg/kg TiO_2_ NPs weekly for 30 days [132].

Results also indicate elevated levels of TNF-α [123,146], and pro-inflammatory IL-6 cytokine [123], and a decrease in anti-inflammatory IL-4 cytokine [146] in reproductive tissues, which indicates a cellular inflammatory response to the NP exposure.

Zhang et al. evaluated male fertility by assessing the offspring of rats treated with Mn_3_O_4_ NPs [110]. The obtained results confirmed that this treatment decreased rats’ fertility and reduced the survival rate of their offspring in a time-dependent manner. For these authors, these results are attributed to changes in reproductive hormones and the decline in sperm quality [110].

In summary, most biochemical and molecular results were concomitant with histological findings. Therefore, despite the many benefits of MONPs, the results of the listed in vivo studies confirm the in vitro studies, emphasizing the possibility that exposure to these NPs could have a detrimental impact on male fertility.

### 4.3. MONPs in Human Reproductive Medicine

The recent approval of MONPs-based technologies in clinical medicine allowed an increase in human living standards and an improvement in mankind’s healthcare conditions through the prevention, early detection, diagnosis, treatment, and follow-up of multiple diseases [153]. However, their usefulness in human reproductive medicine has yet to be proved.

Considering that 50% of infertile couples, the male partner is affected by aberrations in sperm properties, number, vitality, and morphology [154], there is a clear need to develop novel methodologies for the early identification of infertility causes and its treatment. Some research teams have already developed MONP-based approaches that were tested in vitro and in vivo, with promising results. These include methods to reduce oxidative stress induced by cryopreservation [155], improve the proportion of healthy spermatozoa in semen prior to insemination [156], provide movement to sperm with motility deficit [157], protect the fertility of men who are exposed to fertility disrupters [158], and even treat other male associated disorders, such as erectile dysfunction [159].

Although these and other approaches have shown promising results, most of the literature still suggests uncertainty regarding the risk of MONPs in fertility, which may be one of the main reasons why, to date, there are no trials involving this type of engineered NPs for fertility regulation and treatment of male reproductive diseases. Another limiting factor is that only a few studies tried to identify the exact mechanism and pathways induced by MONPs. Current animal experiments also fail to assess pregnancy rates, and the health of offspring, which is the most relevant outcome parameter of fertility [160]. This gap in literature allows the speculation around the hazard posed by MONPs, which could prevent the translation of the results from the lab to the clinical applications [161]. NPs represent a valuable tool to alleviate much of the suffering arising from many reproductive difficulties and disorders, but further work is required to determine if these NPs can fulfill the needs in reproductive health. Human clinical reproductive trials may help accelerate the commercial availability of these new alternatives.

## 5. Conclusions and Future Perspectives

The increased application of MONPs in many industries and scientific fields has made these materials highly present in the environment, resulting in an increased risk of human exposure. Additionally, evidence that keeps emerging suggests that MONPs interfere with the male reproductive system at many biological levels.

The results presented in this review from both in vitro and in vivo studies prove that MONPs can interfere with the male reproductive system, and these results should not be ignored. The collected data show that this reproductive toxicity is achieved due to the MONPs’ ability to interfere with cell molecules and reproductive hormones, which often results in DNA damage and altered gene expression. It was also reported that MONPs induce oxidative stress in germ cells, which affects their number, quality, morphology, and activity. At the organ level, MONPs can cross the BTB and accumulate in the testis, resulting in many histological alterations in tissues of the reproductive system. Since the normal physiological processes that occur in the male reproductive system are highly complex and vulnerable, the interference of MONPs at any level can be deleterious and impair male fertility. Whether these harmful effects are reversible or not is still unclear and should be investigated in further research. How these alterations affect pregnancy and offspring is still an unresolved issue and should be addressed in future studies.

In the studies presented, the only conditions considered to evaluate the reproductive toxicity of MONPs were concentration and duration of exposure. However, the size and surface area are two crucial physical properties that affect how MONPs interact with cells and thus greatly determine the cytotoxicity of NPs. In addition, current studies generally focus on individual alterations but fail to establish a relationship between them. This may be partly the reason why the exact mechanism of nanotoxicity is not yet fully elucidated. Therefore, future studies should make a more in-depth examination of the molecular mechanisms of NPs and MONPs, in particular in reproductive toxicity and the interaction between each reported alteration. In addition, the in vivo studies are of significant heterogeneity, mainly due to the difference in the route of administration and the highly variable administered doses and exposure times. All of these factors can potentially be a source of toxicity that may influence the outcome of the studies. In some cases, unrealistically high concentrations of MONPs were used in cell culture and animal studies, which obviously results in cytotoxicity. Those studies lead to discouraging results that affect the accurate estimation of the reproductive health risks and hinder clinical translation.

It is reasonable to conclude that there are still difficulties in evaluating the reproductive toxicity of MONPs and in understanding exactly how they interact with the male reproductive system. The results summarized in this review reinforce the need for further studies with uniform protocols to obtain solid results with real implications in humans.

## Figures and Tables

**Table 1:** In vitro studies of adverse effects of MONPs on mammalian male germ cells. The conditions where the main findings were observed are indicated in brackets.

MONPs	Characteristics	Concentration and Exposure Time	Cell Type	Parameters	Main Findings	Reference
Cerium oxide	**Formula:** CeO_2_ **Size:** ~7 nm **SA:** 400 m^2^/g **Shape:** Ellipsoidal crystallites	0.01, 0.1, 1, 10 µg/mL 1 h	Spermatozoa (Human)	- Sperm vitality; - DNA damage; - Uptake of NPs	- Sperm viability higher than the normality threshold—58% - Increased DNA damage (≥0.01 µg/mL); - Accumulation of NPs at the plasma membrane, particularly along the flagellum, without internalization	[108]
Iron oxide	**Formula:** Fe_3_O_4_ **Size:** 40 nm **Shape:** spherical	0.192 mg/mL 30, 45, and 60 min	Spermatozoa (Boar)	- Motility and kinetics	- No effects on sperm motility	[109]
Manganese oxide	**Formula:** Mn_3_O_4_ **Size:** ~ 20 ± 4.1 nm **Shape:** irregular sphere-like morphology	0, 5, 10, 20 µg/mL 6 and 24 h	Sertoli Cells (Rats)	- ROS production; - MMP and apoptosis;	- Increase in ROS (5 µg/mL, 24 h); - Alterations in the mitochondrial membrane integrity and increase in the apoptotic rates (≥5 µg/mL, 24 h)	[110]
Titanium oxide	**Formula:** TiO_2_ **Size:** ~30–90 nm **Zeta potential:** −27.3 mV	1, 10, 100 µg/mL 0, 3, 6 h	Spermatozoa (Bufallo)	- Viability; - Acrosomal and plasma membrane integrity; - Capacitation; - Acrosome-reaction; - DNA fragmentation; - Uptake of NPs	- Viability decrease (100 µg/mL, 3 and 6 h); - Decrease in the integrity of the plasma membrane (≥1 µg/mL, 6 h) and acrosomal membrane (100 µg/mL, 6 h); - Increase in capacitation (≥10 µg/mL, 6 h); - Increase in acrosomal reaction (≥1 µg/mL, 3 and 6 h); - Increased DNA fragmentation (≥10 µg/mL, 6 h); - Uptake of NPs mainly in the plasma membrane and sperms’ head	[111]
	**Formula:** TiO_2_ **Size:** ~21 nm **Shape:** spherical **Zeta potential:** −124.55 ± 13.20 mV **HS:** 115.2 ± 11.3 nm **Purity:** >99.5% **PDI:** 0.19	0.1, 1, 10, 100 µg/mL 24 h	Spermatocytes and Sertoli cells (Mouse)	- Viability; - Apoptosis; - Uptake of NPs - Cytoskeleton; - Migration ability; - Phagocytic activity	- Cell viability was not affected; - Increase in the early apoptosis ratio for both cells and in the late apoptosis ratio for Sertoli cells (100 µg/mL); - Dose-dependent uptake of the nanoparticles, mainly in the cytoplasm; - Disordered microtubules (spermatocytes) and microfilaments (Sertoli cells); - Decreased migration ability of spermatocytes (100 µg/mL); - Weakened phagocytic capacity of Sertoli cells (100 µg/mL)	[112]
	**Formula:** TiO_2_ **Size:** ~21 nm **Shape:** partly irregular and semispherical	1, 10 µg/L 15, 30, 45, 90 min	Spermatozoa (Human)	- Viability; - Motility characteristics; - DNA damage; - ROS production	- Cell viability was not affected; - Increase in progressive and nonprogressive sperm (1, 10 µg/L for ≥ 45 min); - Increase in DNA damage (1, 10 µg/L for ≥ 30 min); - Increase in ROS production (1, 10 µg/L for ≥ 15 min)	[113]
Zinc oxide	**Formula:** ZnO **Size:** ~50 nm **Shape:** amorphous	10, 100, 500, 1000 µg/mL 45, 90, and 180 min	Spermatozoa (Human)	- Viability	- Increase in cell death (≥100 µg/mL, 180 min and ≥ 500 µg/mL, ≥ 45 min)	[114]
	**Formula:** ZnO **Size:** ~70 nm **Shape:** spherical **Dispersion:** polydisperse **Surface roughness:** high (22.9 nm)	0, 5, 10, 15, 20 µg/mL 3, 6, 12, and 24 h	Leydig and Sertoli cells (Mouse)	- Viability; - ROS production; - Uptake of NPs; - MMP and apoptosis; - DNA damage	- Decreased viability in both cell types (≥15 µg/mL, ≥6 h); - Increase in ROS production (≥10 µg/mL, ≥6 h) - Accumulation and uptake of nanoparticles’ aggregates in the cytoplasm and nucleus; - Loss of MMP and apoptosis increase (≥15 µg/mL, 6–12 h); - DNA leakage with an increase in chromosome breaks or loss (≥15 µg/mL, ≥12 h)	[115]
	**Formula:** ZnO **Size:** 177 nm **Shape:** spheroid or ellipsoid **Zeta potential:** −27.4 ± 1.0 mV **Purity:** >97%	0, 0.04, 0.08, 0.4, 0.8, 4, 8, 16 µg/mL 24 h	Spermatocytes and Sertoli cells (Mouse)	- Viability; - Oxidative stress indexes (ROS, GSH, MDA) of both cell types; - Membrane permeability, MMP and cytochrome c of Sertoli cells; - TNF-α and Erk1/2 levels of Sertoli cells; - Connexin-43, occludin, claudin-5, ZO-1 expression of Sertoli cells; - DNA damage of spermatocytes; - Cell cycle analysis (cyclin E2, cyclin A2, CDK2) of spermatocytes	- Decrease in cell viability (≥8 µg/mL); - Increase in ROS and MDA levels and decrease in GSH (8 µg/mL); - Increase in membrane permeability with decrease in MMP (8 µg/mL), but no significant changes in cytochrome c (8 µg/mL); - Increase in TNF-α and phosphorylation of Erk1/2 (8 µg/mL);- Decrease in claudin-5, occludin, ZO-1 and connexin-43 expression (8 µg/mL); - Increase in p-Chk1, p-Chk2 and ϒ-H2AX expression but decrease in APE1 (8 µg/mL) but DNA damage can be partly rescued by antioxidants; - Increase in cyclin E2, cyclin A2, CDK2 expression with an increase of cell numbers in the S phase (8 µg/mL)	[116]
	**Formula:** ZnO **Size:** 20–40 nm **Shape:** spherical **HS:** 75 nm	0–200 µg/mL 1, 4, and 12 h	Leydig cells (Mouse)	- Viability; - Cell morphology; - Uptake of NPs; - Apoptosis; - Oxidative stress indexes (SOD, CAT); - Steroidogenesis-related genes expression (StAR, P450scc); - Antioxidant enzyme related gene (SOD); - Testosterone levels in cells’ supernatant	- Decrease in cell viability (≥2 µg/mL, ≥1 h); - Loss of normal morphology (≥5 µg/mL, 4 h); - Randomly dispersed agglomerates of NPs in the cytoplasm, autophagosomes, autolysosomes, mitochondria and in nuclear membranes (50 µg/mL, 4 h); - Apoptosis increase (5 or 20 µg/mL, 4 h); - Increase in SOD (1, 5 µg/mL, 4 h and 5, 20, 50 µg/mL, 12 h), CAT (1, 5, 20 µg/mL, 4 h and 5, 20 µg/mL, 12 h) activity; - Increase in StAR (1, 5 µg/mL, 4 h and 1 µg/mL, 12 h) and P450scc expression (1, 5 µg/mL, 4 h); - Decrease in SOD mRNA (1, 5 µg/mL, 4 h); - Increase in testosterone production (2 µg/mL, 12 h)	[117]
	**Formula:** ZnO **Size:** 30 nm **Zeta potential:** 38.25 ± 1.06 mV **HS:** 66.36 ± 0.93 nm	0, 2, 3, 4, 8 µg/mL 24 h	Leydig cells (Mouse)	- Viability; - Oxidative stress indexes (GPx, GSH, SOD, MDA); - Apoptosis-related proteins (cleaved Casp-8 and Casp-3, Bcl-2, Bax); - Autophagy-related proteins (Atg-5, Beclin-1) and LC3-II/LC3-I ratio	- Decrease in cell viability (≥3 µg/mL); - Increase in MDA levels (≥3 µg/mL) and decrease in SOD, GSH (≥3 µg/mL) and GPx (≥2 µg/mL) levels; - Increase in the expression of cleaved Casp-8, Casp-3 and Bax and decrease in Bcl-2 expression; - Increase in LC3-II to LC3-I ratio and Atg-5 and Beclin-1 expression (4 µg/mL)	[118]
	**Formula:** ZnO **Size:** 88 nm **SA:** 12 m^2^/g **Shape:** spherical **Crystal structure:** hexagonal wurtzite **Zeta potential:** −15 mV (pH = 6) and −55 mV (pH = 12)	1, 5, 8, 10, 20 µg/mL 6 and 12 h	Spermatogonia(Mouse)	- Viability; - Apoptosis and necrosis; - ROS production; - DNA damage; - Cytoskeleton dynamics; - Nucleoskeleton dynamics; - Nuclei morphological changes	- Decrease in cell viability (20 µg/mL, 12 h); - Cell death by necrosis (20 µg/mL, 12 h); - Increase in ROS levels (20 µg/mL, 6 h and ≥5 µg/mL, 12 h); - Increase in DNA damage (20 µg/mL, ≥6 h); - Interference with microtubule and microfilament protein levels (20 µg/mL for 6 h and 12 h); - Alterations of the basal levels and distribution of the nuclear lamina and nuclear envelope proteins (20 µg/mL, 12 h); - Visible morphological deformities in the cells’ nuclei.	[92]

**Abbreviations:** Atg-5, Autophagy Related 5; Bax, Bcl2-associated X protein; Bcl-2, B cell lymphoma-2; Casp-, Caspase; CAT, Catalase; CDK2, Cyclin Dependent Kinase 2; DNA, Deoxyribonucleic Acid; Erk1/2, Extracellular Signal-Regulated Kinase 1/2; GPx, Glutathione Peroxidase; GSH, Reduced Glutathione; HS, Hydrodynamic Size; MDA, Malondialdehyde; MMP, Mitochondrial Membrane Potential; PDI, Polydispersity Index; P450scc, Cytochrome P450 side-chain cleavage enzyme; ROS, Reactive Oxygen Species; SA, Surface Area; SOD, Superoxide Dismutase; StAR, Steroidogenic Acute Regulatory Protein; TNF-α, Tumor Necrosis Factor Alpha; ZO-1, Zonula Occludens-1.

**Table 2:** In vivo studies of adverse effects of MONPs on the mammalian male reproductive system. The conditions where the main findings were observed are indicated in brackets.

MONPs	Characteristics	Dosage and Exposure Duration	Route of Administration	Animal Model/Tissue/Organ/Fluid	Parameters	Main Findings	Reference
Aluminum oxide	**Formula:** Al_2_O_3_ **Size:** 50 nm	70 mg/kg/day 75 days	Oral	Wistar Rats Testis Prostate Epididymis Sperm Plasma	- Reproductive organs weight; - mtTFA, UCP2 testis levels; - DNA fragmentation; - p53, TNF-α, IL-6 testis levels; - Oxidative stress indexes (GPx, GST, CAT, SOD, GSH, TAC, TBARS, NO); - Steroidogenic enzymes levels (17-KSR, 17β-HSD); - Sperm quality; - Reproductive and thyroid hormones levels (testosterone, FSH, LH, TSH, T3, T4); - Testis histopathology	- Decline in testis and epididymis weight but increase in prostate weight; - Suppression and increase of MtTFA and UCP2 expression, respectively; - Massive DNA fragmentation; - Increase in p53, TNF-α and IL-6 levels; - Decrease in GPx, GST, CAT, SOD, GSH, TAC levels and increase in TBARS and NO levels; - Increase and decrease in 17β-HSD and 17-KSD levels, respectively; - Reduction in sperm quality; - Decrease in testosterone and TSH levels, increase in FSH, LH, T3 and T4 levels; - Degenerative changes in testis	[123]
Cerium oxide	**Formula:** CeO_2_ **Size:** <25 nm **Purity:** >99%	10, 20, 40 mg/kg/day 32 days	Oral	C57BL/6J Mice Testis Epididymis Epididymis Sperm Plasma	- Ce accumulation; - Testis weight; - Sperm quality; - Testis histopathology; - Testicular marker enzymes levels (ACP, G6PD, γ-GT, SDH); - Testosterone and transcription factors genes expression (StAR, P450scc, P450c17, 3β-HSD, 17β-HSD, SF-1)	- Increase of Ce content in testis and in denatured sperm DNA (≥20 mg/kg); - Decrease in testis weight (40 mg/kg); - Reduction in sperm quality (≥20 mg/kg); - Seminiferous tubules damage and apoptosis in interstitial tissue (≥20 mg/kg); - Decreased activities of G6PD, SDH, γ-GT (≥20 mg/kg) and ACP (40 mg/kg); - Decrease in testosterone levels and expression of SF-1, StAR, P450scc, P450c17, 3β-HSD (≥20 mg/kg)	[124]
Iron oxides	**Formula:** Fe_2_O_3_ **Size:** 20 ± 5 nm	5, 10, 20, 40 mg/kg 2 weeks	Intraperitoneal	Mice Testis Epididymis Epididymis Sperm	- Sperm quality; - Testis histopathology	- Reduction in sperm quality (≥5 mg/kg); - Reduction of spermatids and spermatocytes in ST and detachment of spermatogonia and spermatocytes from ST wall	[125]
	**Formula:** Fe_2_O_3_ **Size:** <50 nm	25, 50 mg/kg/week 4 weeks	Intraperitoneal	Albino Mice Testis Epididymis Serum	- Total protein in the testis; - Sperm quality; - Testis and serum LDH and testosterone levels; - Testis histopathology; - Fe accumulation; - Oxidative stress indexes (ROS, MDA, SOD, NO, LPO, PC, CAT, GPx, GSH, vitamin C); - DNA damage and apoptosis (Bax, cleaved-Casp3 and -PARP)	- Decrease in total protein in the testis (≥25 mg/kg); - Reduction in sperm quality (≥25 mg/kg); - Increase in testosterone and LDH levels (≥25 mg/kg); - Detachment, sloughing and vacuolization of ST (≥25 mg/kg); - Increased Fe levels in the testis and in serum (≥25 mg/kg); - Increase in ROS, LPO, PC, SOD, NO, CAT, GPx (≥25 mg/kg), decrease in CAT, GSH (50 mg/kg) and vitamin C (≥25 mg/kg) levels; - Increase in the expression of Bax, cleaved-PARP and -Casp3, confirming DNA damage and apoptosis	[97]
	**Formula:** Fe_3_O_4_ **Size:** 20–30 nm	50, 150, 300 mg/kg/day 4 days	Intraperitoneal	NMRI Mice Epididymis Testis Semen	- Sperm quality; - Testis cell number (spermatogonia, primary spermatocytes, spermatids, Sertoli and Leydig cells); - ST morphometry; - Volume of testis and interstitial tissue	- No significant changes in sperm number, decrease in VCL, VSL, VAP and rapid progressive motility values and increase in the percentage of immotile sperm (300 mg/kg/day); - Reductions in the total number of testicular cells; - Reduction in ST length, volume of the testis and interstitial tissue (300 mg/kg/day)	[126]
	**Formula:** Fe_3_O_4_ **Size:** <50 nm	5 mg/kg/day 79 days	Oral	Wistar Rats Epididymis Sperm Plasma Testis	- Sperm quality; - Reproductive and thyroid hormones levels (testosterone, TSH, FSH, LH, T3, T4); - Activity enzymes related to testosterone production (17β-HSD and 17-KSD activity)	- Reduction in sperm count, motility and increase in abnormal sperm; - Decrease in testosterone and TSH levels, increase in FSH, LH, T3 and T4 levels; - Reduction in 17β-HSD and 17-KSD activity	[127]
Manganese oxides	**Formula:** Mn_2_O_3_ **Size:** ~70 nm	100, 200, 400 mg/kg/day 14 days	Oral	Wistar Rats Testis Epididymis Blood	- Reproductive hormones levels (testosterone, LH and FSH); - Testis cell number (spermatogonia, primary spermatocytes, spermatids, Leydig cells); - Testis histopathology	- Decrease in testosterone, LH and FSH levels (400 mg/kg); - Reduction in testicular cell number (400 mg/kg); - Cellular disruption of ST (≥200 mg/kg), interstitial edema of ST, appearance of vacuoles in epithelium and reduction in cell regulation (400 mg/kg)	[128]
	**Formula:** MnO_2_ **Size:** 25–85 nm	100 mg/kg/week 4 weeks	Subcutaneous	Wistar Rats Testis Epididymis Seminal vesicle Prostate Serum Epididymis Sperm	- Testis cell number (sperm, spermatozoa, spermatogonia and spermatocytes); - Reproductive organs weight; - Reproductive hormones levels (testosterone, E2, FSH); - Sperm quality; - Testis histopathology; - ST morphometry;	- Reduction in testicular cell number; - No difference in the prostate, epididymis and left testicle’s weight; - No significant difference in FSH, E2 and testosterone levels (4th week); - Decrease in sperm number and motility (100% immotile sperm, 4th week); - Fluid accumulation in the interstitial space of germline cells; - Decrease in ST mean diameter	[129]
	**Formula:** Mn_3_O_4_ **Size:** ~20 ± 4.1 nm **Shape:** irregular sphere-like morphology	10 mg/kg/week0, 60, 120 days	Intravenous	Sprague–Dawley Rats Testis Epididymis Sperm Serum	- Mn biodistribution in testis and serum; - Testis morphometry and histopathology; - Reproductive hormones levels (testosterone, LH, FSH); - Oxidative stress indexes (MDA, SOD); - Sperm quality; - Fertility evaluation; - Transcription profiling in the testis	- Increase in Mn content in serum and testis (≥60 days); - Reduction of the thickness of germinative layer (≥60 days) and ST degeneration (120 days); - Decline in testosterone and FSH but increase in LH levels (120 days); - Increase in SOD and MDA levels (120 days); - Increase in sperm abnormalities, decrease in sperm concentration and motility (120 days); - Decrease in fertility and fetal survival rate (120 days); - Upregulation of PPAR-signaling pathway and increased expression of cytochrome P450	[110]
Titanium oxide	**Formula:** TiO_2_ **Size:** 5–6 nm **SA:** 174.8 m^2^/g **HS:** 294 nm **Zeta potential:** 9.28 mV	2.5, 5, 10 mg/kg/day 60 days	Intragastric	ICR Mice Testis Epididymis Epididymis Sperm	- Testis weight; - Sperm quality; - LDH, SODH, SDH, G-6PD, ACP, AKP, TNOS, Ca^2+^-ATPase, Ca^2+^/Mg^2+^-ATPase, and Na^+^ /K^+^ -ATPase levels; - Oxidative stress indexes (ROS, MDA, PC, 8-OHdG); - Testis and epididymis histopathology	- Reduction in testis weight (≥5 mg/kg); - Decline in sperm concentration, motility (≥5 mg/kg) and increase in morphological abnormalities (≥2.5 mg/kg); - Decreased activity of LDH, SODH (≥5 mg/kg), SDH, G6PD, ATPases (≥2.5 mg/kg), and elevated activity of ACP (≥5 mg/kg), AKP and NOS (≥2.5 mg/kg); - Increase in ROS (≥2.5 mg/kg), MDA, PC and 8-OHdG (≥5 mg/kg) levels; - ST degeneration, reduced number of Leydig cells and mature sperm within the lumen, sperm breakages, spermatolysis, androgone fusion and/or pycnosis (≥2.5 mg/kg);	[130]
	**Formula:** TiO_2_ **Size:** ~ 5.5 nm **SA:** 174.8 m^2^/g **HS:** 208–330 nm **Zeta potential:** 9.28 mV	1.25, 2.5, 5 mg/kg/day 6 months	Intragastric	CD-1 Mice Testis Epididymis Sperm	- Reproductive organs weight; - Ti accumulation; - Sperm quality; - Testis and epididymis histopathology; - Cdc2, Cyclin B1, Gsk3-β, TERT, Tesmin, TESP-1, XPD, XRCCI, PGAM1/4 and DMC1 expression	- Decrease in testis (≥2.5 mg/kg) and epididymis weight (≥1.25 mg/kg); - Increase in Ti content in testis and epididymis (≥2.5 mg/kg); - Decrease in sperm number, motility rate and increase in abnormalities (≥1.25 mg/kg); - Pathological changes in the testis and epididymis with NPs agglomerates in the ST and few spermatozoa in epididymis lumen (≥1.25 mg/kg); - Decreased expression of Cdc2, DMC1, TERT, Tesmin, Cyclin B1, XRCC1 and XPD and increased expression of Gsk3-β and PGAM4 (≥1.25 mg/kg)	[131]
	**Formula:** TiO_2_ **Size:** 21 nm	5, 25, 50 mg/kg/week 4 weeks	Intravenous	Wistar Rats Testis Serum	- Ti accumulation; - Oxidative stress indexes (CAT, SOD, GPx, LPO); - CK, testosterone and Casp-3 levels; - Sperm number; - DNA damage and apoptosis; - Testis histopathology	- Accumulation of Ti in the testis (≥5 mg/kg); - Decrease in SOD and GPx and increase in CAT and LPO levels (≥25 mg/kg); - Increase in CK levels and in Casp3 activity (50 mg/kg) but decrease in testosterone levels (≥25 mg/kg); - Decline in sperm count; - DNA damage and apoptosis (≥25 mg/kg); - Disorganized and disrupted ST with NPs aggregates in spermatids, Sertoli and Leydig cells (50 mg/kg)	[132]
	**Formula:** TiO_2_ **Size:** 10 nm	100 mg/kg/day 4 and 8 weeks	Oral intubation	Albino Rats Testis Epididymis Seminal vesicle Prostate gland Epididymis Sperm Serum	- Reproductive organs weight; - Testosterone levels; - Sperm quality; - Testis, epididymis, prostate gland and seminal vesicle histopathology	- Decrease in testis, epididymis (8th week), and seminal vesicle weight (4th week); - Decrease in testosterone levels (≥4th week); - Decrease in sperm motility, concentration and viability with increase of sperm abnormalities (≥4th week); - Interstitial edema and sloughing of SE, pyknosis, karyolysis and karyoschisis in testis; congestion, vacuolation and inflammatory cells infiltration with spermatid coagulum in epididymis; congestion, hyperplasia and desquamation of prostate’s epithelial lining; congestion in seminal vesicle	[133]
	**Formula:** TiO_2_ **Z-average size:** 150 d.nm	0.1, 1, 2, 10 mg/kg/week 4 weeks	Intravenous	C57BL/6J Mice Testis Epididymis Sperm Epididymis Plasma	- Reproductive organs weight; - Sperm quality; - Reproductive hormones levels (testosterone, LH, FSH, GnRH); - Ti accumulation	- No significant changes in the testis and epididymis weight; - Decrease in sperm number (10 mg/kg) and in motile and progressive sperm (≥0.1 mg/kg); - Only testosterone levels were decreased (0.1 mg/kg); - No significant accumulation of Ti in the testis	[134]
	**Formula:** TiO_2_ N/A	100 mg/kg/day 8 weeks	Oral intubation	Albino Rats Epididymis Sperm Serum Blood Testis	- Sperm quality; - Oxidative stress indexes (CAT, GSH, MDA); - Testosterone, Casp-3 and Testin levels; - Testis histopathology	- Decrease in sperm quality; - Decline in the levels of testosterone and GSH and increase in MDA levels, with non-significant effect on CAT; - Activation of Casp3, indicating apoptosis and upregulation of Testin gene; - Interstitial edema and sloughing of the germinal epithelium with apoptotic changes	[135]
	**Formula:** TiO_2_ **Size:** ~10 nm **SA:** 120 m^2^/g **Purity:** >99.8% **Shape:** rhabditiform **Zeta potential:** −20.7 to −3.77 mV	0, 10, 50, 100 mg/kg/day 28 days	Intragastrical	ICR Mice Epididymis Sperm Epididymis Testis	- Reproductive organs weight; - Sperm quality; - Oxidative stress indexes (SOD, MDA); - Testis histopathology	- No significant changes in testis and epididymis weight; - No significant changes in sperm density and increase in sperm malformation (≥50 mg/kg); - Decrease in SOD (100 mg/kg) and increase in MDA (≥50 mg/kg) content; - Disordered and vacuolized spermatogenic cells with reduced number (≥50 mg/kg)	[136]
	**Formula:** TiO_2_ **Size:** 17 nm **SA:** 107.7 m^2^/g **Z-average size:** 218 nm **PDI:** 0.24	63 µg/week 7 weeks	Intratracheal	C57BL/6J Testis Epididymis	- Reproductive organs weight; - Sperm count; - Testosterone levels;	- No significant changes in testis and epididymis weight; - No significant changes in sperm count; - No significant effect on testosterone levels;	[137]
	**Formula:** TiO_2_ **Z-average size:** 150 d.nm	0, 2, 10 mg/kg/week 4 weeks	Intravenous	C57BL/6J Mice Testis Epididymis Sperm Epididymis	- Reproductive organs weight; - Sperm quality; - Ti accumulation	- No significant changes in testis and epididymis weight; - Decrease in sperm number and in motile and progressive sperm (≥2 mg/kg); - No significant accumulation of Ti in the testis	[138]
	**Formula:** TiO_2_ **Size:** ~40 nm	100 mg/kg/day 60 days	Oral gavage	Wistar Rats Testis Epididymis Sperm	- Sperm quality; - Oxidative stress indexes (CAT, SOD, GPx, MDA, GSH, FRAP values); - SE and ST morphometry; - Testis histopathology	- Decline in sperm quality; - Increase in MDA levels, decrease in CAT, SOD, GPx, GSH and FRAP values; - Decline in the diameter of ST and height of SE; - ST with irregular shape, wide interstitial space with reduced number of Leydig cells	[139]
	**Formula:** TiO_2_ **Size:** < 25 nm **Shape:** spherical **Zeta potential:** +2.8 to +5.8 mV **PDI:** 0.822 **HS:** 1492 nm	9.38, 18.75, 37.5, 75 mg/kg/day 35 days	Intraperitoneal	Swiss Mice Testis Serum Epididymis Sperm	- Testis weight; - Sperm quality; - Reproductive hormone levels (testosterone, LH, FSH); - Oxidative stress indexes (SOD, CAT, GSH, MDA); - Testis tissue morphometry; - Testis histopathology	- No significant changes in testicular weight; - Decrease in motile sperm (≥9.38 mg/kg) and in sperm count with an increase in sperm abnormalities (≥18.75 mg/kg); - Decrease in LH (≥9.38 mg/kg) and FSH (75 mg/kg) levels, with no significant changes in testosterone levels; - Reduced activity of SOD (≤37.5 mg/kg), CAT (≥9.38 mg/kg) and GSH (9.38 mg/kg) and increased MDA levels (≥18.75 mg/kg); - Decrease in germinal height (9.38, 37.5, 75 mg/kg) and increase of luminal width (≥9.38 mg/kg); - Increased number of damaged ST, Leydig cell degeneration and necrosis of spermatogenic cells (75 mg/kg)	[140]
Zinc Oxide	**Formula:** ZnO N/A	0, 5, 50, 300 mg/kg/day 35 days	Oral	NMRI Mice Epididymis Testis Epididymis Sperm	- Testis weight; - Sperm quality; - ST histopathology; - SE maturity; - ST and SE morphometry	- Decrease in testis weight (300 mg/kg); - Decrease in sperm number and motility, increase in abnormalities (≥50 mg/kg); - Increase in detached, sloughed (≥50 mg/kg), vacuolized (≥5 mg/kg) and multinucleated ST (300 mg/kg); - SE maturation arrest with abnormal spermatogenesis (≥50 mg/kg); - Decrease in ST diameter and SE height (≥50 mg/kg)	[141]
	**Formula:** ZnO **Size:** 10–30 nm **SA:** 20/30 m^2^/g **Crystal phase:** single **Crystal morphology:** nearly spherical **Density:** 5.606 g/cm^3^ **Purity:** ≥99%	0, 50, 100, 150, 200 mg/kg/day 10 days	Intraperitoneal	Wistar Rats Liver Kidneys Epididymis Sperm Serum	- SOD, GPx, MDA, TAC, TOS levels; - Sperm quality;	- No difference in the levels of SOD and GPx, increase in MDA (≥100 mg/kg) and TOS (200 mg/kg) and decrease in TAC (200 mg/kg) levels; - Decrease in sperm count, viability, normal morphology (≥50 mg/kg) and motility (≥100 mg/kg);	[142]
	**Formula:** ZnO **Size:** 20 nm **SA:** >90 m^2^/g **Color:** white **Crystal morphology:** nearly spherical **Purity:** ≥99%	0, 250, 500, 700 mg/kg/day 7 days	Intraperitoneal	NMRI Mice Testis	- Testis weight; - Testis histopathology	- No alterations in testis weight; - No alterations in the tunica albuginea thickness and no increase in degenerated ST. Decrease in ST and SE diameter (250 and 500 mg/kg). Decrease in the number of A type spermatogonia (≥500 mg/kg), primary spermatocytes (500 mg/kg) and fibroblasts (≥250 mg/kg). Higher number of degenerated cells, and multinucleated spermatids (≥250 mg/kg). No alterations in the number of Sertoli, spermatids, spermatozoa, and B type spermatogonia cells	[143]
	**Formula:** ZnO **Size:** ~ 70 nm **Shape:** spherical **Nature:** crystalline **Dispersion:** polydisperse **Surface roughness:** high (22.9 nm)	0, 1, 5 mg/kg single dose at PND21	Intravenous	CD-1 Mice Epididymis Testis Epididymis Sperm	- SE and ST morphometry; - Sperm morphology	- Reduction in SE thickness (5 mg/kg, PND28 and PND42) but no differences in ST diameter; - Increase in sperm abnormalities (≥1 mg/kg, 49 days after injection)	[115]
	**Formula:** ZnO **Size:** <50 nm **SA:** >10.8 m^2^/g **Purity:** >97%	0, 100, 400 mg/kg/day 12 weeks	Intragastric	Albino Rats Epididymis Testis Epididymis Sperm Serum	- Sperm quality; - Oxidative stress indexes (MDA, CAT, SOD, GPx, GSH); - Testosterone levels; - Expression of enzymes related to testosterone production (3β-HSD, 17β-HSD and Nr5A1); - Testis histopathology	- Decline in sperm motility, viability (≥100 mg/kg) and concentration and increase in sperm abnormalities (400 mg/kg); - Increase in MDA (400 mg/kg), decrease in GSH, GPx, SOD and CAT (≥100 mg/kg) levels; - Reduction in testosterone production (≥100 mg/kg); - Reduction in the expression of 3β-HSD, 17β-HSD and Nr5A1 (≥100 mg/kg); - Increased cell apoptosis, ST damage, sloughing of immature germ cells from ST (≥100 mg/kg)	[144]
	**Formula:** ZnO **Size:** 39.45 ± 19.88 nm **HS:** 447.5 nm **Aggregation:** large and irregular **PDI:** 0.13 nm **Shape:** hexagonal **Zeta potential:** −32.1 mV	300, 2000 mg/kg twice at 24 h interval	Oral	Swiss Mice Liver Epididymis Sperm	- Sperm quality; - Liver ROS and 8-oxo-G levels	- Decline in sperm count (2000 mg/kg), motility, viability (≥300 mg/kg) and increase in aberrant sperm during the maturation phase (2000 mg/kg); - Increase in ROS levels and 8-oxo-G expression (2000 mg/kg)	[145]
	**Formula:** ZnO **Size:** <100 nm **Purity:** ≥99.5% **Color:** white	0, 422 mg/kg/day 4 weeks	Oral gavage	Albino Rats Testis Prostate Serum	- Oxidative stress indexes (MDA, GSH, CAT, SOD); - Testis and prostatic cytokines content (TNF-α, IL-4); - Testis and prostate DNA fragmentation; - Testis and prostate histopathology;	- Elevation of MDA and reduction of GSH, CAT, SOD; - Increase in TNF-α and decrease in IL-4; - Confirmed DNA fragmentation; - Tunica albuginea with congested blood vessels, disorganized ST with cell loss and absence of spermatozoa, SE separated from basement membranes and some germ cells with dark pyknotic nuclei;	[146]
	**Formula:** ZnO **Size:** 50 nm **Shape:** cube **Color:** white **Purity:** 99.99%	100, 200 mg/kg/day 7 and 14 days	Oral gavage	Albino Mice Testis Epididymis Seminal vesicle Prostate Epididymis Sperm	- Reproductive organs weight; - Sperm abnormalities	- Decline in testis and epididymis weight but hypertrophy of seminal vesicle and prostate (≥100 mg/kg, ≥7 days); - Increase in sperm abnormalities (≥100 mg/kg, ≥7 days)	[147]
	**Formula:** ZnO **Size:** 30 nm **Zeta potential:** 38.25 ± 1.06 mV **HS:** 66.36 ± 0.93 nm	0, 100, 200, 400 mg/kg/day 28 days	Intragastric	Kunming Mice Testis Epididymis Serum	- Testosterone levels; - Testis and epididymis histopathology; - Gene expression related to apoptosis (cleaved Casp-3 and -8, Bax, Bcl-2) and autophagy (Atg-5, Beclin-1, ratio LC3-II/LC3-I)	- Decrease in testosterone levels (≥200 mg/kg); - Mildly disorganized ST (200 mg/kg), disintegration of SE, germ cell depletion and reduction in round sperm in the ST (400 mg/kg); - Upregulation of cleaved Casp-8 (≥100 mg/kg), Casp-3 and Bax (400 mg/kg) and downregulation in Bcl-2 (≥100 mg/kg) expression in the testis. Increase in Atg-5, Beclin-1 expression, and LC3-II/LC3-I ratio in the testis (≥100 mg/kg);	[118]
	**Formula:** ZnO **Size:** 30 nm **Shape:** spherical	0, 50, 150, 450 mg/kg/day 14 days	Oral gavage	Kunming Mice Epididymis Testis Testis Sperm Serum	- Reproductive organs weight; - Sperm count; - Testis histopathology; - Zinc accumulation; - Gene expression related to apoptosis (Casp-3, -9 and -12, JNK, Bcl-2/Bax) ER stress (BIP, XBP1s, IRE1α, CHOP) and testosterone production (StAR, cytochrome P450scc); - Testosterone levels	- Increase in testis (150 mg/kg) and epididymis weight (50 and 450 mg/kg); - Low number of sperm in the ST lumen (50 mg/kg), ST degeneration and vacuolization of Sertoli cells (150 mg/kg), Leydig cells vacuolization, absent ST with degenerated and necrotic spermatogenic cells (450 mg/kg); - Zinc accumulation in the epididymis (50 and 450 mg/kg) but not in the testis; - Upregulation of BIP, XBP1s, Casp-12 (450 mg/kg), IRE1α, Casp-3 (≥50 mg/kg), CHOP (≥150 mg/kg) and Casp-9 (150 mg/kg). Downregulation of JNK at 50 mg/kg but upregulation at 150 mg/kg and down-regulation of Bax/Bcl-2; - Decrease in sperm number and testosterone levels (≥150 mg/kg), related to the downregulation of StAR	[90]
	**Formula:** ZnO **Size:** 100 nm	100 mg/kg/day 75 days	Oral	Wistar Rats Testis Prostate Epididymis Sperm Plasma	- Reproductive organs weight; - mtTFA, UCP2 testis levels; - DNA fragmentation; - p53, TNF-α, IL-6 testis levels; - Oxidative stress indexes (GPx, GST, CAT, SOD, GSH, TAC, TBARS, NO); - Steroidogenic enzymes levels (17-KSR, 17β-HSD); - Sperm quality; - Reproductive and thyroid hormones levels (testosterone, FSH, LH, TSH, T3, T4); - Testis histopathology	- Decline in testis and epididymis weight but increase in prostate weight; - Suppression and induction of MtTFA and UCP2 expression, respectively; - Massive DNA fragmentation; - Increase in p53, TNF-α and IL-6 levels; - Decrease in GPx, GST, CAT, SOD, GSH, TAC levels and increase in TBARS and NO levels; - Increase and decrease in 17β-HSD and 17-KSD levels, respectively;- Reduction in sperm count, motility and increase in sperm abnormalities; - Decrease in testosterone and TSH levels, increase in FSH, LH, T3 and T4 levels; - ST with irregular shaped and empty lumina, spermatogenic cells with pyknotic nuclei, few Leydig cells	[123]
	**Formula:** ZnO **Size:** <100 nm **Shape:** rod-like **Zeta potential:** +17 to +20.6 mV **PDI:** 0.729 **HS:** 882.8 nm	9.38, 18.75, 37.5, 75 mg/kg/day 35 days	Intraperitoneal	Swiss Mice Serum Epididymis Sperm Testis	- Testis weight; - Sperm quality; - Reproductive hormones levels (testosterone, LH, FSH); - Oxidative stress indexes (SOD, CAT, GSH, MDA); - Morphometric parameters; - Testis histopathology;	- No significant changes in testis weight; - Decrease in motile sperm, lower sperm number (≥9.38 mg/kg), increase in sperm abnormalities (18.75 and 37.5 mg/kg) and higher testosterone levels (≥9.38 mg/kg); - Decrease in LH (9.38, 18.75 and 75 mg/kg) and FSH (≥37.5 mg/kg) levels; - Reduced SOD and CAT activity but increased MDA activity (≥9.38 mg/kg) with no significant changes in GSH; - Decrease in germinal height (≥9.38 mg/kg) and increase of luminal width (9.38, 37.5, 75 mg/kg); - Increased number of damaged ST, Leydig cell degeneration and necrosis of spermatogenic cells (≥9.38 mg/kg)	[140]
	**Formula:** ZnO **Size:** 80 nm	0, 150, 350 mg/kg 15 days	Oral	Albino Mice Testis Prostate Seminal Vesicle Epididymis	- Testis, prostate, seminal vesicle, and epididymis histopathology	- Mild damage in seminal vesicles and epididymis (150 mg/kg) and severe damage in all tissues of the reproductive system (350 mg/kg)	[148]

**Abbreviations:** ACP, Acid Phosphatase; ALT, Alanine Aminotransferase; AST, Aspartate Aminotransferase; AKP, Alkaline Phosphatase; Bax, Bcl2-associated X protein; Bcl-2, B cell lymphoma-2; BIP, Immunoglobulin-Binding Protein; Casp-, Caspase; CAT, Catalase; Cdc-, Cyclin Dependent Kinase; CHOP, Transcription of CCAAT/enhancer-binding Protein (C/EBP); CK, Creatine Kinase; DMC1, DNA Meiotic Recombinase 1; DNA, Deoxyribonucleic Acid; E2, 17β-estradiol; FRAP, Ferric Reducing Antioxidant Power; FSH, Follicle Stimulating Hormone; GnRH, Gonadotropin-Releasing Hormone; GST, Glutathione S-transferase; GSH, Reduced Glutathione; Gsk3-β, Glycogen synthase kinase 3 beta; GPx, Glutathione Peroxidase; G6PD, Gluco-6-Phosphate Dehydrogenase; HS, Hydrodynamic Size; IL-, Interleukin; IRE1α, Inositol-Requiring Protein 1α; JNK, Jun Kinase; LDH, Lactate Dehydrogenase; LH, Luteinizing Hormone; LPO, Lipid Peroxidation; MDA, Malondialdehyde; mtTFA, mitochondrial Transcription Factor A; NO, Nitric Oxide; Nr5A1, Nuclear Receptor Subfamily 5 group A member 1; PARP, Poly (ADP-ribose) Polymerase; PC, Protein Carbonyl; PDI, Polydispersity Index; PGAM1/4, Phosphoglycerate Mutase 1; PND, Post-Natal Days; P450scc, Cytochrome P450 side-chain cleavage enzyme; ROS, Reactive Oxygen Species; SA, Surface Area; SDH, succinate dehydrogenase; SE, Seminiferous Epithelium; SF-1, Steroidogenic Factor-1; SOD, Superoxide Dismutase; SODH, Sorbitol Dehydrogenase; ST, Seminiferous Tubules; StAR, Steroidogenic Acute Regulatory Protein; TAC, Total Antioxidant Capacity; TBARS, Thiobarbituric Acid-Reactive Substances; TERT, Telomerase Reverse Transcriptase; Tesmin, Testis Expressed Metallothionein Like Protein; TESP-1, Testicular Serine Protease 1; TNF-α, Tumor Necrosis Factor Alpha; TNOS, Total Nitric Oxide Synthase; TOS, Total Oxidant Status; TSH, Thyroid Stimulating Hormone; T3, Tri-iodothyronine; T4, Thyroxin; UCP2, Uncoupling Protein 2; XBP1s, X-Box-Binding Protein 1 splicing; XRCC1, X-Ray Repair Cross Complementing 1; VAP, Average Path Velocity; VCL, Curvilinear Velocity; VSL, Straight Line Velocity; 3β-KSD, 3β-hydroxysteroid dehydrogenase; 8-OHdG, 8-hydroxydeoxyguanosine; 17-KSR, 17-Ketosteroid Reductase; 17β-HSD, 17β-hydroxysteroid dehydrogenase; γ-GT, γ-glutamyl-transpeptidase.

## Data Availability

Not applicable.
